# A Review on Autonomic Functional Assessment in Diabetic Patients

**DOI:** 10.7759/cureus.34598

**Published:** 2023-02-03

**Authors:** Charushila Rukadikar, Atul Rukadikar, Surekha Kishore

**Affiliations:** 1 Physiology, All India Institute of Medical Sciences, Gorakhpur, Gorakhpur, IND; 2 Microbiology, All India Institute of Medical Sciences, Gorakhpur, Gorakhpur, IND; 3 Community Medicine and Family Medicine, All India Institute of Medical Sciences, Gorakhpur, Gorakhpur, IND

**Keywords:** prevalence, who, mortality, morbidity, diabetes, cardiovascular, autonomic function test

## Abstract

In today's world, science has progressed significantly, yet most people are still unaware of diabetes. Lack of obesity, physical work, and lifestyle changes are the main factors. Diabetes is becoming more common all around the globe. Type 2 diabetes may go unnoticed for years, resulting in serious consequences and high healthcare expenses. The goal of this study is to look at a wide range of studies in which the autonomic function of diabetic people has been studied with the help of various autonomic function tests (AFTs). AFT is a non-invasive approach to assessing patients for testing sympathetic and parasympathetic responses to stimuli. AFT findings give us comprehensive knowledge of the autonomic physiology reactions in normal and in autonomic diseases like diabetes. This review will concentrate on AFTs that are scientifically valid, trustworthy, and clinically beneficial, according to experts.

## Introduction and background

Diabetes is a leading cause of morbidity [1] and death [2] across the globe. The name diabetes comes from the Greek word Diabanein, which means “to pass through,” and refers to the increased urine production which can be common in various diseases. The name “diabetes” is commonly used to refer to diabetes mellitus, which roughly translates to “excess sweet urine” (known as “glycosuria”). Diabetes is a very common metabolic disorder that affects almost every organ system in the body. Diabetes is a lifelong or generational disease that is greatly influenced by daily diet, activity, mutations, and stress. Diabetes is expected to impact approximately 300 million people by the year 2025 [3]. Hypoglycemia or hyperglycemia are typical short-term consequences that may need hospitalization [4]. There are currently approximately 40.9 million patients with diabetes mellitus in India and this number is expected to rise to about 69.9 million by the year 2025. This high burden of diabetes is likely to be associated with an increase in associated complications. Autonomic nervous system (ANS) dysfunction is one of the significant complications of diabetes mellitus and this is generally associated with a poor prognosis. Cardiac parasympathetic involvement precedes sympathetic damage. According to the recommendations of the American Diabetes Association (1992), five standard cardiovascular reflex tests are used to assess cardiovascular autonomic function. These include changes in heart rate during deep timed breathing, Valsalva maneuver, and standing up to assess cardiac parasympathetic activity and blood pressure responses to standing up and sustained handgrip to evaluate sympathetic nervous activity [5]. Diabetes has been more prevalent in recent centuries. According to increased fat content, unplanned diet, or genetic mutation on a global scale, diabetes is a serious disease that has been called “the great health challenge of the twenty-first century.” Diabetes, as well as its consequences, has become more common. Type 2 diabetes mellitus (T2DM) is now causing an epidemic across the globe [6]. According to the International Diabetes Federation (IDF), about five million people died worldwide in 2015 because of diabetes and its complications [7]. T1DM prevalence is rapidly increasing, as is the incidence of T2DM among younger people. Cardiac autonomic neuropathy (CAN) is one of the most underappreciated diabetic consequences [8]. Although it is quiet in its early phases, it is a robust predictor of mortality in diabetic patients and poses a significant challenge to all clinicians who treat diabetic patients. Due to a significant risk of silent myocardial ischemia, sudden death, and cardiac arrhythmias, patients with CAN have a five-fold greater risk of mortality. The growing problem of diabetes is indicated not only in the expanding number of patients but also in the rising number of diabetes-related early deaths. In the study by Balcıoğlu et al., CAN be discovered in 76% of the patients out of which sympathetic neuropathy was discovered in 54% of the patients, whereas parasympathetic neuropathy was reported in 22% [9]. Because diabetes is a chronic condition that may last a lifetime or even be passed down from generation to generation, effective therapeutic techniques, with a focus on nutrition, should be provided by a healthcare provider or dietician to control the illness, alleviate symptoms, and avoid complications. Patients should also be well informed about their condition and nutrition, and they should be advised to change their eating habits and prepare nutritious foods by their healthcare providers. Diabetes and its consequences may be avoided with active and efficient nutritional education. Diabetes-related CAN is widespread in both type 1 and type 2 diabetes and causes considerable morbidity and death. The pathophysiological pathways that lead to CAN are complex, and further research is needed. The treatment and diagnosis of illnesses and disorders of the ANS are the focus of autonomic medicine. Autonomic function tests give quantitative data on the activity and integrity of the autonomic nerves, CNS, ganglia, and CNS networks, which are critical for a proper diagnosis. Since the ANS cannot be directly tested, clinical autonomic tests examine an end-organ response to a physiologic stimulus. The electrodiagnostic examination of neurologic diseases requires testing of motor and sensory nerve function, as well as autonomic nerve activity. Electrodiagnostic autonomic testing, unlike motor and sensory nerve conduction investigations, focuses on small-diameter unmyelinated and myelinated nerve fibers. Autonomic dysfunctional recognition, measurements, and localization are important for patients' diagnosis, clinical care, and prognosis in order to enhance their quality of life. For more than three decades at leading facilities, various laboratory assays of parasympathetic and sympathetic responses have been developed, resulting in a significant body of papers and experiences that characterize autonomic physiology in health and autonomic dysfunction in disease. Differences in sweating, blood pressure (BP), and heart rate (HR) are all part of this examination. Furthermore, autonomic testing equipment and procedures have been standardized [10].

Autonomic diseases might be arranged by the clinical condition that is being discussed or by the underlying diagnosis. In any case, there is an overlap. For example, alpha synucleinopathy of the central nervous system or peripheral autonomic neuropathy might be present in a patient with orthostatic hypotension. The present overview is organized by the several forms of autonomic issues seen in clinical practice, where the etiology may or may not be obvious before autonomic testing. The advent of noninvasive electrodiagnostic validated standardized techniques that measure the ANS’s functional integrity has improved the clinical detection of ANS problems. Expert agreement on adequate autonomic test performance has permitted diagnostic and prognostic linkages to many common and unusual autonomic illnesses which continues to enrich our knowledge of their causes.

## Review

Diabetic-associated neuropathy

Diabetic autonomic neuropathy (DAN) is a significant diabetic consequence of either T1DM or T2DM. It may affect every organ in the body and cause a range of symptoms. DAN was a neglected field before the previous two decades, and its frequency was underestimated at that time since it was assumed to be an uncommon or late consequence of diabetes [11]. After ruling out alternative causes of autonomic neuropathy, DAN is described as a malfunction or impairment of the sympathetic and/or parasympathetic ANS in diabetic individuals. Clinical signs of DAN include arrhythmias and sudden death [12] depending on the organ affected. According to studies, the prevalence of DAN in type 1 diabetes ranges from 1% to 90%. The large range of results is attributable to discrepancies in the DAN diagnosis criteria and study methodology [13]. Clear clinical evidence of DAN is uncommon; however subclinical DAN has been observed in individuals with T1D within two years and in patients with T2D within one year [14]. Studies showed that the highest mortality in CAN is due to silent myocardial ischemia, life-threatening arrhythmia, and sudden cardiac death [15]. Furthermore, studies have shown that the presence of DAN reduces the adrenaline response to hypoglycemia in hypoglycemic diabetics. After an exhaustive study of the available literature, the Consensus Panel on Diabetic Neuropathy estimated the prevalence of CAN to be 20%-65% in an unselected population of T1D or T2D patients. Ewing has published a battery of basic non-invasive autonomic testing [16, 17]. Subsets of this assessment are sensitive to ANS malfunction in the sympathetic and parasympathetic nervous systems. In summary, studies have shown that duration of diabetes, age, vascular complications, glycemic control (polyneuropathy, nephropathy, retinopathy, etc.), and comorbidities (e.g., dyslipidemia/hypercholesterolemia, hypertension) were all risk factors for DAN [18]. The pathogenesis of DAN, including CAN, is thought to be similar to that of diabetic peripheral neuropathy and is characterized by an increase in the production of advanced glycation end products (AGE), nutritional shortages, immune-mediated, oxidative stress, hormonal, growth factor inadequacy, and neurovascular insufficiency, as well as a metabolic insult to neurons [19].

Autonomic nervous system (ANS)

To maintain homeostasis, the ANS is made up of networks of neurons that govern multiple organ systems within the body, employing a variety of substrates and signals. ANS is divided into two divisions - sympathetic and parasympathetic nervous systems. The sympathetic component is “fight or flight,” whereas the parasympathetic component is “rest and digest.” It controls smooth muscle, cardiac muscle, and endocrine and exocrine glands, which regulate urine production, blood pressure, thermoregulation, and bowel functions without conscious control throughout an organism's existence [20].

Anatomy and Physiology of ANS

The feedback canters, central control, peripheral effectors, sensory receptors, and reflex conduction routes make up the architecture of the ANS. Furthermore, the endocrine system and the ANS have complicated interconnections [21]. To maintain physiologic homeostasis, the renin-angiotensin system, glucocorticoid, mineralocorticoid responses, antidiuretic hormone, and insulin interact through an increasing number of tissue receptors.

In the cerebral cortex, there are no separate areas of autonomic function. However, sensory information from multiple systems may reach higher cortical areas, where it is processed and translated into efferent autonomic activity. Higher cortical sensory processing includes a physical finding of tachycardia and peripheral vasoconstriction, which signal a “fight-or-flight” reaction, or a vasovagal response (fainting) [22]. The senses of smell, hearing, touch, and sight perceive external stimuli that pose a threat or danger. These signals are delivered to the brainstem, where the hypothalamus and limbic forebrain process reflex reactions. The paraventricular nucleus of the hypothalamus, which contains connections to the sympathetic and parasympathetic nuclei, receives descending information from higher cortical areas. Chronic stress changes these structures and functions, making the stress response more sensitive and habitual [23]. The sympathetic and parasympathetic functions, fluid regulation, temperature regulation, neurohumoral control, and stress reactions are all processed by the hypothalamus, a midbrain area. The hypothalamus regulates sleep, hunger, and sexual function which rely on both cerebral input and sophisticated feedback regulation. The anterior hypothalamus regulates temperature, while the posterior hypothalamus regulates water. The hypothalamic-pituitary axis is a component of the ANS that controls long-term blood pressure and stress responses [24]. The sympathetic and parasympathetic systems are physically and functionally separate divisions of the ANS, which provide some level of nervous input to various tissues. As a result, the frequency of neuronal firing in both systems might increase and decrease. Therefore, tissue activity may be boosted or suppressed. This feature of the ANS boosts its capacity to control tissue function more accurately. Nervous input to specific tissue could rise if there was no tonic action. Both systems innervate many tissues. Because the sympathetic and parasympathetic nervous systems usually have opposite effects which ultimately affect a tissue's function [25]. When faced with a situation that requires full attention - such as deciding whether to “fight or flee” - the brain requires as much oxygen as possible (for processing sensory information), to achieve this state the respiratory passageways dilate to allow as much air as possible to enter the lung and the heart must pump more blood to transport oxygen systematically to the needed organs (e.g., brain, heart and skeletal muscles). The sympathetic (fight or flight) and parasympathetic (rest and digest) activities of the ANS might be seen as opposing each other. The parasympathetic branch has an inhibitory impact on target cells in general, while the sympathetic branch has a stimulating effect.

Sympathetic division: The sympathetic system's preganglionic neurons emerge from the lumbar and thoracic areas of the spinal cord (segments T1 through L2). Most of these preganglionic axons are short and connect to postganglionic neurons in sympathetic ganglion chains. These ganglion chains run parallel along the spinal cord has 22 ganglia. The preganglionic neuron may leave the spinal cord and connect with a postganglionic neuron in the same ganglion from where it emerged. The preganglionic neuron may also move farther rostrally or caudally (upward or downward) in the ganglion chain, synapsing with postganglionic neurons in ganglia at different levels. A single preganglionic neuron may connect with many postganglionic neurons in multiple ganglia [20,26].

**Figure 1:**
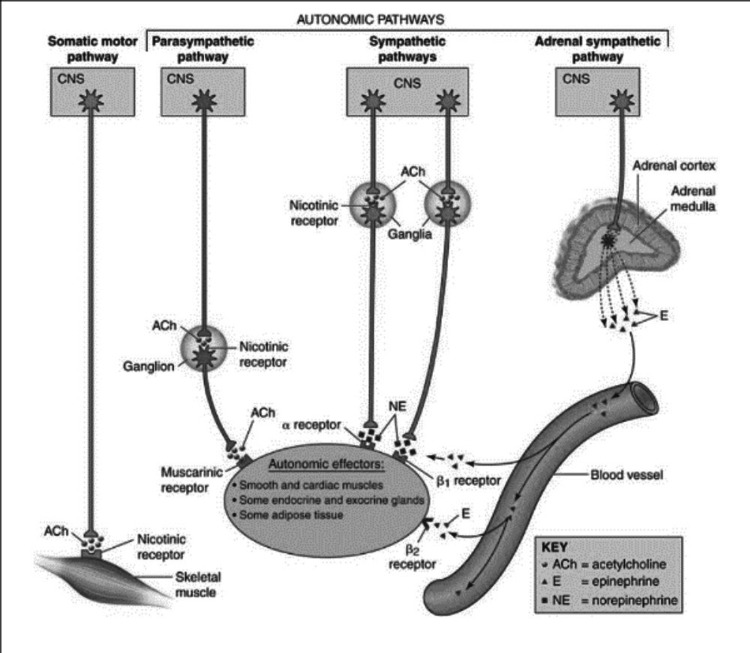
Autonomic nervous system pathways

Parasympathetic division: The cranial part of the parasympathetic nervous system consists of cranial nerves III, VII, IX, and X, as well as pelvic splanchnic nerves that emerge from S2 to S4. The parasympathetic nervous system is organized similarly to the sympathetic nervous system, with preganglionic fibers synapsing with postganglionic fibers that eventually innervate target areas. The preganglionic nerve fibers synapse on ganglia that are adjacent to or embedded inside their respective target locations, resulting in postganglionic neurons that are short [27]. Preganglionic neurons have longer axons than sympathetic neurons, and they synapse with postganglionic neurons inside terminal ganglia that are adjacent to or embedded within effector tissues. The extremely short axons of the postganglionic neurons subsequently send input to the cells of the effector tissue. The cranial nerves allow preganglionic neurons from the brainstem to leave the CNS. Figure 1 represents autonomic pathways [28].

Pathophysiology of Autonomic Dysfunction

The pathophysiology of the ANS is determined by the afflicted region. Anatomically linked and separated areas might both be impacted. Three pathophysiological disorders are often linked with persistent ANS dysfunction in the cardiovascular system. Orthostatic hypotension with supine hypertension, reflex cardiovascular syndromes, and postural orthostatic tachycardia syndrome (POTS), are examples of these conditions. The major characteristics of temperature control are hyperhidrosis or hypohidrosis. Diabetic neuropathy's precise pathogenesis is unknown. Schwann cells, nerve axons, and dorsal root ganglia have all been shown to be affected by dyslipidemia and hyperglycemia. Protein kinase C (PKC) activation, Polyol pathway activation, oxidative stress, and AGEs production have all been implicated in nerve damage and death. These noxious pathways are activated by hyperglycemia and vascular risk factors, and they induce harm to axons, microvascular endothelium, and nerve support cells. However, in type 2 diabetes, attention has recently shifted to dyslipidemia and increased triacylglycerols as a source of non-esterified fatty acids (NEFA). Beta oxidation catabolizes NEFA, resulting in acetyl CoA buildup and conversion to toxic acylcarnitine, which leads to nerve damage. Reactive oxygen species are produced when low-density lipoproteins (LDLs) are oxidized, and receptors for advanced glycation end products, oxidized LDLs, and TLR4 are activated, worsening nerve damage. A growing body of data suggests that in type 2 diabetes, altered sphingolipid metabolism leads to the development of atypical neurotoxic deoxysphingolipids, which can contribute to nerve injury [29]. All types of diabetic neuropathy have this pathogenesis.

Pathology of Disturbed ANS System in Diabetes

**Figure 2:**
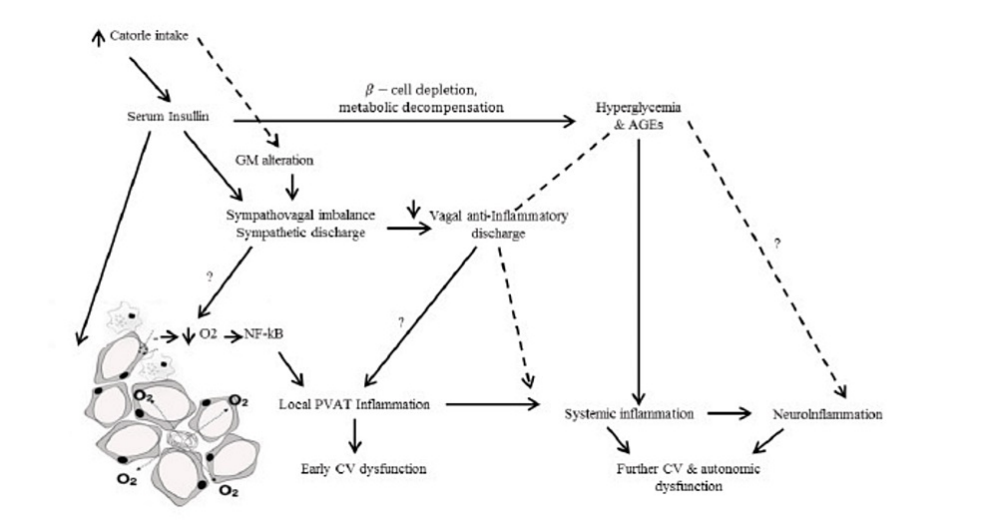
Autonomic dysfunction pathophysiology

Diabetic individuals often have autonomic neuropathy. Significant peripheral neuropathy affects 20% to 40% of insulin-dependent diabetics, resulting in thermoregulatory abnormalities, labile blood pressure, and gastroparesis (possibly due to vagal nerve dysfunction) [30]. This set of issues may need changes to the standard anesthetic protocol, including pretreatment to reduce the risk of cardiovascular support, aspiration of stomach contents, and proactive attempts to avoid hypothermia. Diabetic patients are more stressed and are at a higher risk for perioperative complications, perhaps due to autonomic dysfunction. Distal and sensory predominate nerve fiber degeneration, axonal loss, and endoneurial microangiopathy are the most common peripheral nervous system abnormalities in diabetics. Nerve fibers of both large and small diameters are damaged [31]. Dyck et al. hypothesized that microvascular damage is the most likely source of focal fiber loss and that the combination of these factors seems to be the cause of diffuse fiber loss of distal axonal neuropathies in diabetic patients [32]. However, this argument is oversimplified and fails to explain why hyperglycemia and the duration of diabetes are so important in the disease of diabetes. There's also some debate over whether small fiber involvement in early diabetic neuropathy is more common than large fiber involvement. Patients who did not have clinically obvious neuropathy at the time of nerve biopsy but subsequently exhibited high-grade microangiopathic alterations of endoneurial microvessels producing overt neuropathy, while those who did not have microvessel changes did not acquire neuropathy (Figure 2), according to Malik et al. [33].

ANS Mechanism in Diabetes

Diabetes is the most frequent cause of neuropathy globally, resulting in a broad range of symptoms involving various nerve types and pathogenic processes (e.g., ischemic, metabolic, compressive, and immunologic) [34]. Diabetic autonomic neuropathy is often associated with diabetic DSP, although it seldom occurs alone. Until late in the illness, autonomic symptoms and impairments are typically minor. The pathways that contribute to diabetic DSP are yet unknown. The pathophysiology of diabetic sensorimotor polyneuropathy (DSP) is widely acknowledged to be multifaceted, comprising complicated interactions between glycemic management, age-related neuronal attrition, duration of diabetes, and other variables like blood pressure, cholesterol levels, and weight [35]. Hyperglycemia, glucotoxicity, and poor insulin signaling all function in tandem with other risk factors to activate various biochemical pathways that impact cellular metabolism. These modifications cause structural changes such as Wallerian degeneration, segmental demyelination, and microangiopathy, as well as neuronal apoptosis in the dorsal root ganglia, affecting myelination and resulting in nerve fiber damage or loss [36]. Hyperglycemia causes an increase in mitochondrial free radical generation [37], which in combination with a lack of antioxidant defenses, activates additional harmful pathways. These additional mechanisms include increased formation of AGEs [38], downregulation of the soluble receptor for AGEs [39], activation of polyol aldose reductase signaling with an accumulation of protein kinase C [40], cyclooxygenase 2 activation, activation of poly(ADP ribose) polymerase, endothelial dysfunction, peroxynitrite, and protein nitration [41], and altered function of the Na+/K+-ATPase pump [42]; all having a direct impact on neuronal activity, membrane permeability, endothelial function, and mitochondrial function. Table 1 reveals the effects of the ANS on various organs.

**Table 1:** Effect of two branches of ANS

Sympathetic effect	Parasympathetic effect	Organ
Increased rate and pressure	Lower rate and pressure	Heart
Variable depending on the neurotransmitter	No innervation	Veins
Ejaculation	Erection	Male genitalia
Increased activity	No innervation	Sweat glands
Far focus (lower curvature)	Near focus (increased curvature)	Lens
Dilation	Constriction	Pupil
Decreased filtration rate	Increased filtration rate	Kidneys
Dilation of respiratory passages	Constriction of respiratory Passage	Lungs
High in viscosity	Serous	Salivary gland secretion
Decreased motility	Increased motility	Gastrointestinal
Dilation	No innervation	Arteries to skeletal muscle
Variables depending on the neurotransmitter	Relaxation	Vascular smooth muscle

Mechanism of Autonomic Dysfunction in Diabetes

The existence of neuropathy was attributed to the aberrant cardiovascular autonomic function in early reports by Ewing, based on the discovery of a reduced nerve conduction velocity and longer terminal latency in patients with an abnormal heart rate response to the Valsalva test. In individuals with cardiovascular autonomic dysfunction, diabetic peripheral neuropathy is common, although there is a lower-than-expected concordance with a varied association between them. Increased intima media thickness at the region of the decreased distensibility, baroreceptors, left ventricular hypertrophy, altered cardiac vagal function, and endothelial dysfunction are all non-neural abnormalities that potentially contribute to the autonomic dysfunction observed in patients with diabetes mellitus. These anomalies are, interestingly, strongly linked to the existence of (micro-) albuminuria, which is thought to be a manifestation of endothelial dysfunction or vascular damage in diabetes mellitus [43]. Furthermore, in both T1D and T2D patients, autonomic dysfunction is exacerbated when diabetes is accompanied by (micro-)albuminuria. Indeed, autonomic dysfunction might account for the increase in higher mortality linked to (micro-)albuminuria in diabetes mellitus. In contrast, since autonomic dysfunction declines renal function worsening renal illness, diabetes mellitus may account for the increased mortality in individuals with diabetes mellitus compounded by autonomic neuropathy [44]. Another study of 684 diabetic individuals has confirmed the tight relationship between vascular disease and autonomic dysfunction. The prevalence and severity of cardiovascular autonomic dysfunction were linked to microvascular illnesses such as retinopathy and microalbuminuria, as well as erectile dysfunction [45]. However, the authors involved in the Hoorn Study suggested that cardiovascular autonomic dysfunction could not explain the increased cardiovascular mortality in the general population or diabetic subjects because they discovered that microalbuminuria and cardiovascular autonomic dysfunction had a correlation but no independent association with cardiovascular mortality [46]. However, their proposed cardiovascular autonomic dysfunction score is not validated measurement and the interdependence of cardiovascular autonomic and vascular function may have led to an over-correction in their models. Increased sensitivity to deadly arrhythmias, similar to post-myocardial infarction patients, is another way by which autonomic dysfunction may worsen prognosis. As reflected in diabetics that have a high percentage of unexplained fatalities [47]. Finally, the idea that autonomic neuropathy is caused by prolonged hyperglycemia is inconsistent with recent studies that found higher rates of type 2 in non-diabetic children. Diabetics as non-diabetic offspring of non-diabetics. These individuals were apparently not exposed to prolonged hyperglycemia. Rather, dysautonomia was associated with symptoms of metabolic syndrome. Microvascular injury in this study (elevated urinary albumin levels excretion). Diabetes causes excessive blood glucose and high amounts of lipids in the blood, such as triglycerides, which damage neurons and microvascularity resulting in autonomic neuropathy.

Autonomic function tests (AFTs)

AFTs assist the physician in diagnosing the existence of dysautonomia, its severity, and distribution; and since they are quantitative, whether it is improving or worsening. This might assist the doctor in devising the best effective treatment plan. The first goal is to use non-invasive quantitative assays to assess the degree and cardiovagal distribution, sudomotor, and adrenergic dysfunction. The second goal is to determine if autonomic failure is minimal or restricted. The third goal is to identify and assess orthostatic intolerance. The laboratory will be able to identify orthostatic hypotension (OH) using a head-up tilt (HUT) assessment. An increased heart rate response may now be used to diagnose more subtle changes in HUT, such as POTS. The fourth goal is to track dysautonomia progression. The research laboratory allows the practitioner to quantify whether the situation is improving or worse, as well as the pace of change. For example, Parkinson's disease has a moderate pace of change, but multiple system atrophy (MSA) has a considerably faster rate of change. Lastly, the fifth goal is to track therapy response. When used in clinical studies, autonomic testing is very beneficial. It is feasible to offer several autonomic failures and assess if specific medication improves or worsens outcomes [48].

Cardiovascular Autonomic Testing

The Valsalva technique, standing up (30:15 ratio), blood pressure responses (postural BP change); deep breathing (minimum-maximum heart rate), and prolonged handgrip are the five tests utilized in the standard cardiovascular autonomic assessment. Some have previously argued that cardiac parasympathetic integrity indicates cardiac parasympathetic integrity, but the autonomic networks involved in these responses are complex, and blood pressure fluctuations may be more severe. abnormal only with more extensive (extracardiac) sympathetic nerve damage All five tests involve sympathetic and parasympathetic innervation, and the division into sympathetic and parasympathetic tests is clinically useful but not accurate. It represents all the complex underlying physiological processes.

Valsalva Technique Used in Cardiovascular Tests

After 15 seconds of silence, the person blows into a mouthpiece at 40 mmHg. During the procedure, the patient's heart rate normally increases, followed by rebound bradycardia. The smallest R-R interval ratio during the maneuver to the longest R-R period immediately after the move is then calculated. The calculations produce the outcome of the Valsalva ratio which is commonly expressed as the average of three Valsalva motions.

Heart rate response to deep breathing: The participant sits quietly for a few moments before breathing deeply and evenly at a rate of 6 breaths per minute. The maximum and lowest heart rates during each breathing cycle are measured, and the difference between the two is averaged across three cycles.

Standing up causes a rise in heart rate: The individual rests calmly on a sofa before rising unassisted. Normally, after beginning to stand, there is an instantaneous rise in heart rate that peaks around the 15th beat, then relative bradycardia ensues at the 30th beat. The 30:15 ratio compares the greater R-R interval around the 30th beat to the smallest R-R interval around the 15th beat.

Continuous handgrip raises blood pressure (BP): The BP is monitored every minute using a handgrip dynamometer, keeping the handgrip at 30% of maximal voluntary contraction for a maximum of 5 minutes. The difference in diastolic blood pressure immediately before handgrip release and just before commencing is used to determine responsiveness.

Standing up causes a rise in blood pressure: A typical sphygmomanometer is used to take the subject's blood pressure when they are laying down and again when they stand up. The difference in systolic BP is used to calculate the postural BP change [49].

Historical overview

The pulse intensity and timing, cutaneous and pallor flushing, and changes in physiological secretions have all been used by physicians to measure health and sickness since antiquity. In the early twentieth century, when Langley coined the phrase “autonomic nervous system,” many researchers use tilting boards to study the impact of gravity on blood circulation [50]. The BP responses to the Valsalva maneuver were first documented in the 20th century in the settings of autonomic illness and heart failure [51,52]. In the 1980s, noninvasive methods to assess blood pressure were developed, including digital finger assessment, which progressed from a research tool to a clinical application. These methods use photoplethysmographic technology to indirectly detect blood pressure as a shift in the infrared light passing through the finger as a measure of blood flow. The blood pressure curve mimics the intra-arterial waveform, allowing cardiovascular adrenergic responses on a moment-by-moment basis [53]. With the growing recognition of autonomic disorders as severe health issues and the identification of a wide range of specific autonomic ailments, diagnostic methods have developed after these foundational breakthroughs [54]. Both the Autonomic Nervous System Journal and Clinical Autonomic Research (later called or renamed Autonomic Neuroscience: Basic and Clinical) were first published in 1979 and 1991, respectively, in the developing area of autonomic medicine. Since its inception in 1990, the American Autonomic Society has conducted an annual international conference. Affiliation with the United Council for Neurologic Subspecialties (UCNS) has given subspecialty certification in autonomic illnesses since 2009. The development of novel pharmacologic and other successful therapies for people with autonomic problems has supplemented these resources. This cooperation has resulted in common knowledge and testing approaches to autonomic diseases [55].

Common clinical questions

Clinically, patients with autonomic issues might be difficult. They may exhibit a wide range of symptoms affecting several organ systems. The term “dysautonomia” is often used when accessing a person's symptoms including any element of autonomic function. It's important to note that dysautonomia is a general category rather than a particular diagnosis, far as “weakness” is to a neuromuscular subspecialist. To establish an accurate diagnosis, more probing is required, as well as an intelligently obtained full autonomic history, appropriate autonomic tests, and physical examination [56,57]. Autonomic testing is used to determine if autonomic dysfunction exists, how widespread it is, and how severe it is. Autonomic testing may also uncover hyperfunction autonomic failure and particular patterns that are linked to certain illnesses. The autonomic testing findings may help distinguish between life-threatening and non-life-threatening disorders, as well as identify possibly curable autonomic problems. When autonomic testing findings are aberrant, they may help with an early diagnosis, monitoring clinical development, and assessing therapeutic response. Autonomic testing may offer objective proof that a significant autonomic disorder is not present when the findings are normal.

Autonomic testing standardization

The autonomic test development and labs have resulted in a wide range of autonomic function assessment methodologies. For research findings to be accessed in clinical practice, a consensus has arisen on autonomic disease classifications and testing methodologies [58-60]. 

Cardiac autonomic function and heart-rate variability in diabetes

Simple noninvasive cardiovascular reflex testing made it clear that autonomic deterioration in diabetes was not only more prevalent than previously assumed, but also ubiquitous throughout the body. Because it was discovered in people with long-term diabetes, autonomic neuropathy was formerly assumed to be a late consequence. However, when more advanced tools for testing autonomic function were available, it was clear that autonomic nerve dysfunction occurs considerably earlier during diabetes. As a result, a sensitive test to identify early neuropathic alterations is required. By examining short-term alterations in the cardiac cycle as a mirror of sympathetic and parasympathetic integrity, standard tests of cardiac autonomic function (Valsalva maneuver, deep breathing, 30:15 ratio) may reveal anomalies before the development of clinical symptoms. These tests were originally intended to identify people based on whether they had neuropathy; however, more recent research has sought to rate the degree of neuropathy. Heart-rate variability (HRV) for short-term (5 min) measurement has been suggested as a marker for autonomic neuropathy. However, there is debate about whether the recording should be done standing, sitting, or laying down, as well as the optimal technique for assessing HRV. Ambulatory ECG monitoring (Holter monitor) has been used to evaluate HRV as a test of autonomic function in addition to identifying anomalies in heart rhythm. Despite the potential for future research, no more trials using this approach in diabetic people have been conducted. The ability to evaluate autonomic function during regular everyday activities while being monitored for 24 hours is a once-in-a-lifetime opportunity. It may be used to track the evolution of diabetes. The notion that measuring 24-hour HRV is an appropriate approach for detecting changes in cardiac autonomic function in diabetes mellitus was investigated in this paper [61]. Table 2 reveals different autonomic tests to address various autonomic diseases.

**Table 2:** Autonomic testing indicators

Diagnosis	Autonomic Testing Addresses Clinical Issues
Autonomic failure	Distinguish from benign syndromes or symptoms Evaluate familial dysautonomia Evaluate its distribution, severity, and presence.
Ganglionopathy	Evaluate autoimmune autonomic ganglionopathy Evaluate the distribution, presence, and severity of the autonomic failure
Neurodegenerative disorders	In Lewy body dementia, assess autonomic failure Examine autonomic failure in its purest form Distinguish multiple-system cerebellar atrophy caused by other types of cerebellar ataxia Differentiate between Parkinson's disease and multiple system atrophy Assess autonomic failure in individuals with MSA (multiple system atrophy) In Parkinson's disease, assess autonomic dysfunction
Regional autonomic failure	Examine for the existence, distribution, and severity of autonomic failure that is more widespread
Orthostatic intolerance syncope	Differentiate between neurogenic and non-neurogenic orthostatic hypotension Examine the function of the baroreflex Examine the existence, severity, and timeliness of the problem
Heat intolerance	Examine the Ross syndrome In Sjögren syndrome, assess small fiber neuropathy. Examine if anhidrosis is present, how severe it is, and where it is found.
Peripheral polyneuropathy	Diagnosis and quantification of distal SFN (small fiber neuropathy) Assess autonomic fiber involvement in peripheral neuropathy. Evaluate diabetic autonomic neuropathy Evaluate leprosy Determine the severity of Lambert Eaton myasthenic syndrome Examine inflammatory demyelinating neuropathy Evaluate paraneoplastic autonomic neuropathy Examine amyloid autonomic neuropathy symptoms. Evaluate Guillain-Barré syndrome Axonal sensory and autonomic neuropathies Evaluate Chagas disease
Hyperadrenergic states	Evaluate autonomic dysreflexia Assess the Morvan syndrome Evaluate baroreflex function Evaluate autonomic storms

CAN in diabetes mellitus

Diabetes-related CAN causes changes in vascular dynamics and HR by damaging autonomic nerve fibers that innervate blood vessels and the heart. CAN is known to influence a variety of organ systems and is a leading cause of morbidity and death in diabetic individuals [62,63]. CAN is an “impairment of cardiovascular autonomic regulation in individuals with established diabetes after eliminating alternative causes,” according to the CAN Subcommittee of the “Toronto Consensus Panel on Diabetic Neuropathy” [64]. Intraoperative cardiovascular liability, exercise intolerance, silent myocardial infarction, orthostasis, and resting tachycardia are only a few of the clinical symptoms of CAN. It is a profoundly disabling condition that significantly reduces the quality of life and life expectancy in diabetic patients [65]. Table 3 presents different autonomic reflexes performed regularly [66].

**Table 3:** Tests of cardiovascular autonomic reflexes are performed regularly (CARTS) ECG is for electrocardiogram; HRV stands for heart rate variability, and bpm stands for beats per minute

Test	Technique	Normal response and values
Heart rate Response to standing	During the continuous ECG observation, the R-R interval is assessed at beats 15 and 30 after standing.	Tachycardia is usually followed by reflex bradycardia. The ratio of 30:15 should be more than 1.03.
Systolic blood pressure response to standing	The supine subject's systolic blood pressure is measured. After 2 minutes, the patient gets up and the systolic blood pressure is taken.	The normal response is a fall of less than10 mm Hg; borderline is a fall of 10-29 mm Hg; abnormal is a fall of more than 30 mm Hg with symptoms.
Beat-to-beat HRV	ECG measures heart rate while the patient breathes in and out at 6 bpm, synchronized with a metronome, while the patient is supine.	A variation in heart rate of more than 15 beats per minute is considered normal, whereas less than 10 beats per minute are considered abnormal. In patients aged 20-24, the lowest normal value for the expiration-inspiration ratio of the R-R interval is 1.17; this value declines with age.
Heart rate response to Valsalva maneuver .	The individual forcefully exhales into the mouthpiece of a manometer to a pressure of 40 mm Hg for 15 seconds, during ECG monitoring	During strain, healthy persons have tachycardia and peripheral vasoconstriction, as well as overshoot bradycardia and an increase in blood pressure. The longest to shortest R-R ratio is usually more than 1.2.
Isometric exercise and diastolic blood pressure	To determine a maximum, the participant squeezes a handgrip dynamometer. The grip is then pressed at a maximum of 30% for 5 minutes.	An increase of more than 16 mm Hg in the opposite arm is considered typical for diastolic blood pressure.

Table 4 shows few studies by previous authors on the use of AFTs used in diabetes.

**Table 4:** Autonomic function test study on diabetes patients

Author	Study or statement	References
Metwalley et al.	A prominent consequence of T1D is CAN. They examine the function of the cardiac ANS in children with type 1 diabetes, as well as its relationship to other demographic, laboratory, and clinical variables. The participants in this crosssectional research were sixty children with type 1 diabetes. They found that sympathetic and parasympathetic autonomic dysfunctions are widespread in children with type 1 diabetes, especially in those who have had diabetes for a longer time and have microvascular problems.	[66]
Pop-Busui et al	Diabetes-related CAN, according to him, is caused by a complicated interplay between glycemic management, illness duration, diastolic and systolic blood pressure, and aging-related neuronal death. Hyperglycemia is assumed to be the main reason, triggering a series of complicated processes and pathways that cause toxic glycosylation and oxidative stress, eventually leading to dysfunction and neuronal death	[15]
Mitchell et al.	The first heart rate response to standing was tested in 38 T1D kids. In certain cases, the authors noticed aberrant testing (a flat or slow increase in heart rate when standing). They also discovered that diabetic kids had a decreased mean “maximum R-R/minimum R-R ratio” in 15% of the cases, suggesting autonomic neuropathy.	[67]
Ewing et al.	543 diabetic patients were tested utilizing five noninvasive basic autonomic tests. Heart rate tests were found to be abnormal 40% of the time, while blood pressure testing was found to be abnormal less than 20% of the time, according to the authors. They also divided them into three categories: early dysfunction (15%), definite dysfunction (18%), and severe dysfunction (22%).	[49]
Young et al.	In seventy-nine youngsters with type 1 diabetes (aged 16 to 19 years), researchers looked at peripheral and autonomic nerve function. The authors found that 72% of the participants had peripheral neuropathy and 31% had abnormal cardiac parasympathetic tests.	[68]
Ziegler et al.	For five years, 32 individuals with T1D (ages 12–36) were evaluated prospectively. At diagnosis and after three, twelve, twenty-four, forty-eight, and sixty months, the authors found anomalies in motor and sensory nerve conduction velocities, aberrant pupillary function, and heart rate fluctuation during deep breathing and at rest.	[69]
Boysen et al.	Twenty children with type 1 diabetes were tested for autonomic function using blood pressure, heart rate, and deep breathing. The patients were also tested utilizing power spectral studies of HRV in the low- and high-frequency bands, as well as baroreflex sensitivity. They found aberrant cardiorespiratory reflexes in 75 percent of patients with atleastoneite and 60 percent of healthy controls. In 30% of patients, they found diminished baroreflex sensitivity together with aberrant HRV, but not in the control group.	[70]

## Conclusions

India is known as the world's diabetes capital. In recent years, most young people have developed diabetes because of a lack of physical activity, stress, and an irregular diet. All day-to-day tasks are carried out by modern-looking machines to get the job done Although simple, this complicates diabetes management. The goal of autonomic testing is to improve the sensitivity and specificity of autonomic failure identification. Clinical autonomic testing has certain limitations. The noninvasive technique is reasonable, although it is not without flaws. For example, beat-to-beat BP devices may occasionally generate false indications; therefore, judgment is crucial when interpreting autonomic testing. Because autonomic testing is a developing and dynamic discipline, rules will and should evolve as well. These findings have paved the way for rapid advancements in autonomic neurology, which have the potential to improve patients' lives.
